# Advancements in Molecular Diagnosis and Pharmacotherapeutic Strategies for Invasive Pituitary Adenomas

**DOI:** 10.1002/iid3.70098

**Published:** 2024-12-17

**Authors:** Dingkai Xu, Ling Wang, Maohua Zheng

**Affiliations:** ^1^ Department of Neurosurgery The First Hospital of Lanzhou University Lanzhou China; ^2^ Department of Endocrinology Liangzhou Hospital Wuwei Gansu China

**Keywords:** biomarker, invasiveness, miRNA, oncogene, pituitary adenoma

## Abstract

**Background:**

The overwhelming majority of pituitary tumors consist of pituitary adenomas (PAs), which have recently also been termed pituitary neuroendocrine tumors (PitNETs). Clinically significant PAs occur in approximately one in every 1000 individuals, while other types of pituitary tumors, such as craniopharyngiomas and pituicytomas, are significantly less common. Although PAs are generally benign, a subset of them exhibits malignant‐like biological traits. They tend to infiltrate and grow aggressively into adjacent tissues and organs, including the dura mater, cavernous sinus, and sphenoid sinus. This invasive behavior often results in the destruction of the normal anatomical architecture of the sella turcica and skull base. Clinically, such tumors are classified as invasive PAs (IPAs), emphasizing their aggressive and destructive nature.

**Objective and Significance:**

Currently, the diagnostic indicators for IPAs frequently suffer from suboptimal sensitivity and specificity. The invasiveness assessment of PAs lacks a definitive gold standard and instead serves as a predictive tool, with a greater number of indicators met suggesting a higher likelihood of invasiveness. Consequently, a comprehensive approach that integrates imaging, pathological, molecular biological, and other disciplinary metrics is crucial for accurate evaluation. Despite surgery being the primary treatment modality for IPAs, their malignant‐like behavior complicates complete resection, resulting in lower resection rates and heightened postoperative recurrence, necessitating multiple surgeries. Therefore, adjunctive drug therapy is often necessary for IPA patients. Preoperative drug therapy can shrink tumor size, facilitating resection and postoperative recovery, mitigating hormone imbalances, delaying recurrence, and enhancing patients' quality of life.

**Conclusions:**

This article comprehensively reviews the diagnostic criteria for assessing the invasiveness of PAs in the domains of imaging, pathology, and molecular biology, provides an overview of the current research status of drug therapy for these conditions, and deepens our insight into the biological and therapeutic aspects of the tumor microenvironment in PAs.

## 1 Introduction

In the fifth edition of the WHO Classification of Endocrine Tumors and Central Nervous System Tumors, published in 2022, pituitary adenomas (PAs) have been redefined as pituitary neuroendocrine tumors (PitNETs), thereby assigning an oncology classification to these predominantly benign neoplasms [1]. PAs exhibit a wide range of sizes, hormonal secretion profiles, and varying invasiveness. Classified based on hormonal hypersecretion, PAs are divided into functional and nonfunctional subtypes. Functional PAs, including prolactin (PRL)‐secreting adenomas (also called prolactinomas, accounting for approximately 50% to 55% of secretory adenomas), growth hormone (GH)‐secreting adenomas (also called somatotropinomas, accounting for approximately 20% to 30%), adrenocorticotropic hormone (ACTH)‐secreting adenomas (also called corticotropinomas, accounting for approximately 5% to 15%), gonadotropin (GN)‐secreting adenomas (also called gonadotropinomas, relatively uncommon, accounting for 5% to 15% of cases), and thyroid‐stimulating hormone (TSH)‐secreting adenomas (also called thyrotropinomas, extremely rare, accounting for less than 1% of cases), primarily cause excessive secretion of pituitary hormones, leading to clinical manifestations like amenorrhea, developmental delays, gigantism, acromegaly, Cushing's syndrome, and sexual dysfunction (Table 1 [2]). In contrast, nonfunctional pituitary adenomas (NFPAs) may remain asymptomatic initially but can later cause headaches due to tumor expansion and compression of adjacent brain tissue, particularly the optic chiasm [3].

**Table 1:** The subtypes of PAs and their associated diseases.

Subtypes	Hormone hypersecretion	Percentage	Diseases	Symptoms
Prolactinomas	Prolactin	50%−55%	Hyperprolactinemia	Women: menstrual irregularities, infertility, galactorrhea Men: erectile dysfunction, decreased libido, infertility, gynecomastia Tumor mass effect: headache, visual impairments
Somatotropinomas	Growth hormone	20%−30%	Gigantism (pre‐puberty) Acromegaly (post‐puberty)	Excessive bone growth, facial deformation, enlarged joints, skin thickening, diabetes, heart problems
Corticotropinomas	Adrenocorticotropic hormone	5%−15%	Cushing's syndrome	Central obesity, moon face, buffalo hump, purple striae on skin, hypertension, osteoporosis
Gonadotropinomas	Follicle‐stimulating hormone Luteinizing hormone	5%−15%	Usually nonfunctional, but may cause sex hormone abnormalities	Precocious puberty, sexual dysfunction, infertility Tumor mass effect: headache, visual impairments
Thyrotropinomas	Thyroid‐stimulating hormone	< 1%	Central hyperthyroidism	Heat intolerance, excessive sweating, palpitations, increased appetite, weight loss, irritability

Both functional and nonfunctional PAs can demonstrate invasive capabilities, traversing the sella turcica floor and dura mater to infiltrate regions like the sphenoid sinus, cavernous sinus, and suprasellar area [4]. Consequently, PAs are further categorized based on their biological behavior into noninvasive adenomas, invasive PAs (IPAs), and pituitary carcinomas [5]. Although histologically these three categories may not be entirely distinguishable, they exhibit distinct growth patterns and behaviors. Noninvasive adenomas grow in an expansive manner, often surrounded by a pseudocapsule, and maintain certain boundaries. In contrast, IPAs exhibit an infiltrative growth pattern, invading adjacent structures. A diagnosis of pituitary carcinoma is only confirmed in cases where there is both intracranial and extracranial metastasis [5]. It is crucial to emphasize that IPAs, despite their invasive nature, remain histologically and biologically benign tumors. Clinically, IPAs are often large adenomas, attributable to their heightened proliferation rate and rapid growth. Notably, NFPAs frequently attain considerable sizes, even reaching the classification of giant adenomas, by the time clinical symptoms emerge [6]. The interval between symptom onset and diagnosis is notably shorter for patients with IPAs compared to those with noninvasive adenomas [7]. Moreover, IPAs demonstrate higher incidences of tissue necrosis, apoplexy, and cystic alterations than noninvasive adenomas [6]. Currently, surgical resection is the primary treatment for PAs, but complete tumor removal is achieved in only 66%−78% of cases [8]. When surgery and chemotherapy are unsuccessful, radiotherapy is often selected to treat PAs, albeit with risks to healthy tissue, such as visual loss, hypopituitarism, and cerebrospinal fluid leaks [9]. This clinical challenge has prompted researchers to explore new markers and therapies. As our understanding of the tumor microenvironment (TME) evolves, immunotherapy emerges as a promising option for drug‐resistant or recurrent PAs [10]. The TME, arising from the interplay between tumor cells and the host, is a complex setting composed of fibroblasts, myofibroblasts, endothelial cells, immune cells, and the extracellular matrix (ECM), influencing tumor proliferation, invasiveness, and angiogenesis [11]. Current research emphasizes immune cells within the TME, including myeloid cells (e.g., tumor‐associated macrophages, dendritic cells) and lymphocytes (e.g., T and B cells), collectively termed tumor microenvironmental immune cells [12]. Despite limited studies on PA immunity, this review also discusses current immunological research to highlight the potential of immune molecules in predicting prognosis and guiding clinically targeted therapy for PAs.

Currently, there exists no unified standard for precisely defining and diagnosing IPAs. The primary basis for diagnosing these tumors relies on a comprehensive evaluation encompassing preoperative imaging, intraoperative exploration, postoperative pathological diagnosis, and molecular biological assessment, all aimed at elucidating tumor growth patterns and biological behavior.

## 2 Preoperative Neuroimaging Assessment

Neuroimaging plays a pivotal role in determining the invasiveness of PAs, particularly before surgery. It primarily assesses whether the adenoma has infiltrated surrounding bone structures, the cavernous sinus, sphenoid sinus, nasopharyngeal cavity, nasal cavity, and other adjacent areas [13]. On X‐ray plain films, IPAs can exhibit destruction of surrounding bone structures, such as the sella floor and dorsum sellae. Computed tomography (CT) scans further reveal the extent of infiltration toward the parasellar region. Magnetic resonance imaging (MRI) preoperatively provides a clearer visualization of the invasion of PAs toward neighboring structures from three different planes [13, 14]. Early neuroimaging examinations, when combined with the morphological and secretory functions of PAs, can aid in predicting the direction of growth and diagnosing the invasiveness of PAs at an early stage. CT examination is useful for observing bone resorption, while MRI assesses the tumor's morphology and the extent of invasion by examining whether the tumor boundary extends beyond the pituitary fossa and invades surrounding tissues [15]. As PAs grow upwards, they may invade the diaphragm sellae and enter the suprasellar cistern. Lateral growth, on the other hand, may involve the cavernous sinus, resulting in displacement, narrowing, or even disappearance of normal cavernous sinus structures.

Currently, MRI stands as the most effective auxiliary examination for predicting tumor invasion in PAs [16]. Both MRI plain scan and enhancement scans offer clear visualization of PAs, their relationship with surrounding structures, as well as tumor apoplexy and cystic changes. The presence of the following signs indicates infiltrative growth of PAs: The tumor breaches the sella floor and protrudes into the sphenoid sinus; the normal morphology of the cavernous sinus is lost, with outward bulging of its edges and indistinct boundaries between the cavernous sinus and the tumor, indicating cavernous sinus invasion. The early enhancement of tumor signals is also suggestive; the diameter of the encircling internal carotid artery narrows or its branches are affected; abnormal signals with irregular edges are observed in the bone of the clivus [5, 17]. Furthermore, when PAs infiltrate the adjacent brain parenchyma, a high level of suspicion for PA infiltration should be maintained [17]. Additionally, imaging indicators can be synergistically used with specific subtypes of PAs to enhance diagnostic accuracy. The subtype of PAs is a crucial determinant of their biological behaviors [18]. For instance, GN‐PAs (especially in elderly patients) are typically noninvasive, whereas sparsely granulated GH‐PAs, Crooke cell adenomas, silent ACTH‐PAs, PRL‐PAs occurring in males, and pituitary‐specific transcription factor 1 (Pit‐1) positive PAs secreting multiple hormones tend to be invasive. Therefore, combining imaging findings with the aforementioned PA subtypes can further clarify whether a PA is invasive or not. Pituitary apoplexy represents a critical and emergent complication that can arise in patients with IPAs. Its intricate etiology is postulated to stem from rapid tumor expansion, which triggers intratumoral ischemia, necrosis, and subsequent hemorrhage. Additionally, the proliferation of irregular blood vessels within the tumor can give rise to fragile sinuses prone to rupture and bleeding [19]. Clinically, this condition presents abruptly with severe symptoms like intense headaches, sudden vision loss, nausea, vomiting, and in severe cases, even altered consciousness, posing a grave risk to patients' lives [20]. Imaging techniques, notably MRI, are instrumental in detecting signs of intratumoral hemorrhage, offering vital insights that guide clinical diagnosis and subsequent treatment strategies.

The invasive nature of IPAs often results in compression of the optic nerve and optic chiasm, causing visual field defects and gradual vision loss [21]. Visual field monitoring serves as an early warning system, promptly detecting any alterations in visual function, which is invaluable for assessing tumor progression and the effectiveness of treatment. Advances in medical imaging technology have significantly enhanced the sophistication of visual field monitoring techniques for IPAs. High‐resolution MRI and CT scans now offer unprecedented clarity in visualizing the intricate anatomical relationship between the tumor and adjacent structures, particularly the extent of compression on the optic nerve and optic chiasm [22]. Additionally, functional MRI techniques, including DTI (diffusion tensor imaging) and fMRI (functional MRI), have emerged as powerful tools for assessing the structural integrity and functional capacity of optic nerve fiber bundles, providing a more holistic view of visual function and facilitating targeted monitoring [23].

Despite advancements in imaging technology, including 3.0 T MRI, there are inherent limitations in accurately assessing the degree of invasiveness of PAs [24]. Certain PAs, despite appearing large and potentially invasive on imaging, may actually be encapsulated within a distinct cyst, sparing adjacent tissues from true invasion and permitting complete resection. Additionally, when IPAs ascend, their morphology can closely resemble that of adenomas with expansive growth, rendering them undistinguishable as a definitive marker of invasiveness [25]. Consequently, relying exclusively on imaging metrics may erroneously prompt surgeons to adopt overly extensive surgical approaches for PA removal. Ultimately, surgical observations and subsequent pathological examinations remain indispensable for an accurate assessment of PA invasiveness.

The Knosp grading system is one of the most commonly used imaging scoring systems for PAs, primarily used to assess the likelihood of cavernous sinus invasion by large PAs. This system evaluates the relationship between PAs and the cavernous sinus by measuring the connection between the adenoma and the diameter of the C4 and C6 segments of the internal carotid artery on coronal MRI of the cavernous sinus (Table 2 [26, 27, 28]). The Hardy–Wilson classification system is also a commonly used grading method in the radiological assessment of PAs. It is primarily used to evaluate the expansion of adenomas, their growth upward or downward toward the diaphragma sellae or sellar floor, and their extension beyond the sella turcica. This system is divided into two parts: grading the expansion of adenomas and grading the suprasellar extension of adenomas (Table 3 [29, 30, 31]).

**Table 2:** The Knosp grading system provides a structured approach to assessing the invasion of PAs into the cavernous sinus, aiding in clinical decision‐making and treatment planning.

Grade	Description
0	Tumor is located inside the inner tangent line
1	Adenoma extends beyond the inner tangent line but does not exceed the median line
2	Tumor extends beyond the median line but does not exceed the outer tangent line
3	Tumor extends outside the outer tangent line
3A	Tumor extends above the cavernous sinus to outside the outer tangent line
3B	Tumor extends below the cavernous sinus to outside the outer tangent line
4	Internal carotid artery within the cavernous sinus is completely surrounded by the tumor

**Table 3:** The Hardy–Wilson classification system is commonly used in the radiological assessment of PAs. It evaluates both the expansion of the adenoma and its suprasellar extension.

Assessment of adenoma expansion		Assessment of suprasellar extension of adenoma	
Grade	Description	Grade	Description
0	Microadenoma, confined within the sella turcica with intact sella floor	A	Adenoma involving only the suprasellar cistern
I	Tumor < 10 mm, with only enlargement of the sella floor but no bony destruction	B	Adenoma involving the floor of the third ventricle, causing upward convexity
II	Tumor ≥ 10 mm, with overall or localized enlargement of sella turcica, intact sella floor	C	Adenoma involving the anterior one‐third of the third ventricle
III	Adenoma ≥ 10 mm, enlargement of the sella turcica with focal bony destruction, partial tumor protrusion beyond the sella	D	Asymmetric upward growth, adenoma protruding into the intracranial space, reaching the level of the foramina of Monro
IV	Diffuse adenoma spread, diffuse destruction of sella turcica structure, adenoma invading surrounding structures such as cavernous sinus, optic nerve, temporal lobe	E	Asymmetric lateral growth, invading the cavernous sinus

## 3 Intraoperative Evaluation

During the surgical procedure, under the meticulous scrutiny of the surgical microscope, it is crucial to meticulously observe whether there is any encroachment of adenoma tissues into the adjacent structures, inclusive of the dura mater, bone, cavernous sinus, cranial nerves, blood vessels, and brain tissue [32]. The presence of invasion is typically discernible by a roughened texture in the affected area. It is important to clarify that although PAs can exhibit expansive growth in various directions, even traversing the diaphragm sellae and adopting a dumbbell shape through the sella turcica foramen, those that grow solely in this dumbbell configuration without infiltrating surrounding tissues are not classified as invasive adenomas. Furthermore, the expansive nature of these adenomas can lead to chronic compression, leading to thinning or even defects in the sellar floor bone. Nevertheless, thinning or destruction of the sellar floor alone does not conclusively indicate adenoma invasion. Rather, a definitive diagnosis of invasion necessitates the confirmation of dura mater involvement or pathological examination revealing adenoma cell infiltration within the adjacent tissues [33].

## 4 Postoperative Dural Pathology Evaluation and Diagnosis

The pathological examination subsequent to surgery serves as a pivotal objective criterion for diagnosing IPAs. By scrutinizing the dural tissue procured during the surgical procedure, pathologists can ascertain the presence or absence of adenoma cell infiltration. In the realm of pathology, several conventional markers are utilized to evaluate the aggressiveness of malignant tumors, including mitotic figures, nuclear atypia, hyperchromatic nuclei, cellular necrosis, and cellular pleomorphism [34]. Advanced microscopic studies have uncovered notable differences between invasive and noninvasive PA specimens, with the invasive group exhibiting heightened rates of nucleolus formation, elevated nucleus‐to‐cytoplasm ratios, and increased mitochondrial counts [5, 7, 35]. These findings underscore the utility of these three indicators in predicting the invasive nature of PAs. Additionally, immunohistochemical analyses have revealed augmented expression levels of MMP‐9 and vascular endothelial growth factor (VEGF) in specimens displaying higher nucleolus frequency, nucleus‐to‐cytoplasm ratios, and mitochondrial abundances, reinforcing the notion that the ultrastructural characteristics of IPA cells are intimately linked to their invasive growth patterns. Nevertheless, it is widely acknowledged that there exists a subtle morphological overlap between invasive and noninvasive PAs, rendering conventional histopathological examination a challenging means to differentiate between the two PAs [36]. While some research has noted the occurrence of pleomorphic tumor cells and nuclei, mitotic figures, as well as capsular invasion in recurrent PAs, these features are not consistently present in IPAs and hence cannot be relied upon as reliable indicators of invasiveness or recurrence potential. Enhancing the detection rate of IPAs through pathological examination of peritumoral tissue is a valuable approach, yet the routine practice of intraoperative sampling from multiple sites poses significant challenges and heightens the risk of missed diagnoses. The discrepancies observed among various studies underscore the potential limitations of relying solely on histological features to assess PA invasiveness. Consequently, it is imperative to augment this methodology by integrating other diagnostic modalities to bolster detection accuracy.

## 5 Molecular Biology‐Guided Diagnosis of IPAs

Advancements in molecular biology have illuminated intricately linked to PA invasiveness, including chromosomal abnormalities, alterations in tumor suppressor genes and oncogenes, proliferation cell antigens, and microRNAs (miRNAs). A comprehensive transcriptome analysis has unveiled distinct gene expression patterns in invasive NFPAs compared to their noninvasive counterparts [37]. Notably, the localized suppression of immune responses and perturbations within the TGF‐β signaling pathway have emerged as key factors contributing to the enhanced invasiveness of PAs [37].

### 5.1 Genetic Changes and Invasive Growth in IPAs

#### 5.1.1 Oncogenes and Their Roles in IPAs

The pituitary tumor‐transforming gene‐1 (*PTTG1*) has garnered significant attention for its overexpression in various endocrine‐related tumors, particularly in pituitary, thyroid, breast, ovarian, and uterine malignancies. Importantly, a robust correlation has been established between elevated *PTTG1* levels and tumor invasiveness, positioning *PTTG1* as a pivotal marker gene closely tied to tumor metastasis [38]. Elevated PTTG expression is thought to directly stimulate cellular proliferation and foster chromosomal instability, thereby enhancing the tumor's invasive potential. Current understanding suggests that *PTTG* plays a dominant role in the initiation, progression, invasive growth, and even the transition of PAs to pituitary carcinoma. The hyperactivity of *PTTG* in the cell cycle results in abnormal, recurrent, and unequal divisions without proper cytokinesis, leading to the formation of aneuploidies and the presence of multinucleated cells within pituitary tumors [39]. Consistent with this, previous investigations have documented a positive association between nuclear *PTTG* expression and tumor recurrence, underscoring its potential as a marker of heightened proliferation in PAs [40]. Furthermore, a meta‐analysis has revealed a potential link between *PTTG* expression and both tumor invasiveness and microvessel density in PAs, suggesting that assessing *PTTG* levels could serve as a useful tool in predicting the malignancy grade of these tumors [41].

Apoptosis emerges as a pivotal process in the development of neoplastic lesions, with *Bcl‐2* playing a central role as an apoptosis‐related oncogene initially identified in human B‐cell follicular lymphoma. The overexpression of *Bcl‐2* is known to extend tumor cell survival, inhibit apoptosis, and enhance tumor dissemination and invasiveness [42]. The *Bcl‐2* family comprises a complex interplay of regulators, divided into two opposing functional groups: those that inhibit apoptosis, like *Bcl‐2*, and those that promote it, such as *Bax* [42]. In investigating intrasellar IPAs, a notable elevation in Bcl‐2 expression has been observed compared to noninvasive PAs, with Bax expression inversely correlated to Bcl‐2 levels [43]. Furthermore, an immunohistochemical study revealed that Bcl‐2 protein expression was markedly reduced in PRL‐PAs and NFPAs, but elevated in GH‐PAs. Conversely, Bax protein expression was significantly decreased in recurrent PAs [44]. These findings suggest that apoptosis‐related proteins like Bcl‐2 and Bax are intricately linked to hormone function and local control in PAs, highlighting their potential significance in tumor progression and behavior.

Somatic mutations in the *GNAS* gene are highly prevalent in sporadic GH‐PAs, with up to 50% of these tumors harboring such mutations [45]. These mutations predominantly occur at codons 201 and 227, resulting in impaired GTPase activity of the G protein subunit α (Gsα) encoded by *GNAS*. This, in turn, activates adenylate cyclase and elevates cyclic adenosine monophosphate (cAMP) levels, a mechanism that resembles the defects observed in patients with multiple endocrine neoplasia syndromes (MAS). Due to the paternal imprinting of *GNAS* in the pituitary gland, only mutations occurring on the maternal allele can lead to the development of PAs [46]. Through a series of signaling pathways, *GNAS* mutations ultimately promote the hypersecretion of GH and the proliferation of GH cells. While the relationship between *GNAS* mutations and the aggressive growth of PAs remains incompletely understood, studies have shown that GH‐PAs with *GNAS* mutations often exhibit unique clinical features, including older patient age, relatively smaller tumor size, lower tendency for aggressive growth, and a histologically dense‐grained morphology [47].

Over the past years, research has shown that somatic defects in *ubiquitin‐specific protease 8* (*USP8)* codons 718–720 are a frequent genetic cause of Cushing's disease, found in up to 62% of corticotropinomas [48]. *USP8* encodes ubiquitin carboxyl‐terminal hydrolase 8, a deubiquitinase involved in protein quality control. Cushing's disease‐associated *USP8* variants disrupt its interaction with 14‐3‐3 proteins, leading to enhanced deubiquitinase activity and increased EGFR recycling, which boosts *POMC* transcription [49]. Initially, corticotropinomas harboring *USP8* variants were considered to exhibit a benign phenotype, based on initial observations that they tended to be smaller compared to wild‐type tumors and typically manifested as overt Cushing's disease rather than silent corticotropinomas [50]. Furthermore, research groups noted higher rates of clinical remission among patients with *USP8* variants [50, 51]. The potential of USP8 as a therapeutic target was also suggested, as inhibitors of USP8 were shown to decrease cell proliferation and ACTH secretion in mouse corticotropinoma‐derived AtT‐20 cells [52]. However, contrary findings emerged from other studies, which reported that *USP8* variants were detected in larger tumors and were linked to increased recurrence rates and/or earlier recurrence times [53].

#### 5.1.2 Tumor Suppressor Genes and Their Impacts on IPAs

*TP53*, a crucial tumor suppressor gene, frequently undergoes mutations in cancer cells, often resulting in heightened nuclear expression of its encoded p53 protein. This increase is typically due to reduced protein degradation mechanisms [54]. Suspicion of *TP53* mutations arises when a substantial proportion of tumor cells exhibit p53 expression, given p53's pivotal role in regulating cell proliferation, apoptosis, and maintaining genomic stability. Notably, p53 expression has been implicated in the aggressive behavior of pituitary tumors, with high p53 levels correlated with cavernous sinus invasion in PAs [55]. *TP53* mutations significantly influence the invasive potential of tumors. A higher proportion of *TP53* variants are associated with stronger tumor invasion abilities [56]. Surprisingly, pathogenic *TP53* mutations were observed at an elevated frequency in functional corticotroph macroadenomas and invasive adenomas, representing a substantial portion of these tumor types [57]. These *TP53* mutations were linked to more aggressive tumor characteristics, posing significant challenges in disease management and prognosis [57]. Furthermore, *TP53* mutations, which are rare and occur in both functioning and silent PAs, are associated with poor clinical outcomes in Cushing's disease, and constitute a poor prognostic factor despite their low frequency, as evidenced by both univariate and multivariate survival analyses [58]. A machine learning approach identified loss of heterozygosity (LOH) as the most predictive variable for aggressive, treatment‐refractory PAs, surpassing *TP53* with an accuracy of 0.88 [59]. Notably, widespread chromosomal LOH causes significant aneuploidy particularly in corticotroph PAs, serving as a novel and highly accurate biomarker for predicting treatment‐refractoriness in IPAs [59].

The *PTEN*‐encoded protein acts as a phosphatase, responsible for degrading PIP3, a lipid generated by PI 3‐Kinase. By degrading PIP3, PTEN effectively counterbalances the activation of the oncogenic PI3K/AKT/mTOR signaling pathway, exerting profound effects on tumor cell cycle progression, apoptosis, tumor invasiveness, and angiogenesis [60]. Consequently, the loss of *PTEN*'s tumor suppressor function is a common phenomenon across various tumor types, including neuroendocrine tumors. A recent investigation revealed intriguing findings regarding PTEN expression in patients with PAs. When compared to a healthy control group, patients with both invasive and noninvasive PAs exhibited a notable upregulation of PTEN expression. However, a crucial distinction emerged: patients with IPAs had significantly lower PTEN levels compared to those with noninvasive PAs [61]. This disparity underscores the pivotal role of PTEN in assessing and predicting the prognosis of elderly PA patients. Furthermore, PTEN expression was found to be reduced in PAs compared to normal pituitary tissues, with an even more pronounced decrease observed in the invasive group versus the noninvasive group. This observation suggests a strong association between PTEN expression levels and tumor invasiveness [62].

### 5.2 Epigenetic Changes and Invasive Growth in IPAs

#### 5.2.1 DNA Methylation

DNA methylation is closely related to the genesis, development, and invasiveness of PAs. High DNA methylation levels are found in invasive and large PAs, with DNA methyltransferase overexpression. CpG site methylation differences in promoters can distinguish PAs from normal pituitary tissue. Histone modifications are associated with increased p53 expression and prolonged progression‐free survival in PAs. Many cell growth and signaling genes show altered methylation in PAs, including cell cycle regulators, signal transduction components, apoptotic regulators, and pituitary developmental signals [63]. Furthermore, elevated methylation levels within the promoter regions of genes such as potassium voltage‐gated channel, Shaker‐related subfamily, beta member 2 (KVβ2), O‐6‐methylguanine‐DNA methyltransferase (MGMT), echinoderm microtubule‐associated protein‐like 2 (EML2), RAS homolog family member D (RHOD), homeobox B1 (HOXB1), NNAT, and P16 suppress the expression of these genes, thereby modulating the proliferation of PAs [64]. Therefore, deeper exploration into the molecular mechanisms underlying DNA methylation in IPAs holds promise for novel diagnostic and therapeutic approaches to the disease.

#### 5.2.2 MiRNAs

MiRNAs constitute a class of small, naturally occurring noncoding RNAs (ncRNAs) that primarily regulate gene expression post‐transcriptionally by either directly cleaving mRNAs or inhibiting protein synthesis. They also have the potential to function as oncogenes or tumor suppressor genes. Research has established a connection between abnormal miRNA expression and the development of PAs [65]. A miRNA profiling study revealed significant alterations in the expression levels of 18 upregulated and 36 downregulated miRNAs in NFPA patients compared to healthy individuals [66]. Notably, the target genes of these differentially expressed miRNAs were predominantly associated with axonogenesis and cancer‐related pathways [66]. One particular miRNA, hsa‐miR‐486‐5p, has emerged as a promising biomarker for both the diagnosis and prognosis prediction of NFPAs. Additionally, the significant decrease in the levels of miRNA‐26b, miRNA‐138, miRNA‐206, and miRNA‐let‐7e in the peripheral serum of NFPA patients compared to healthy controls suggested their potential involvement in the initiation and progression of these tumors [67]. He et al. [68] further investigated the role of miR‐448 in PA tissues and cell lines, discovering its downregulation in these contexts. Notably, enhancing the expression of miR‐448 significantly inhibited the proliferation and migration of PA cells while promoting apoptosis, highlighting its crucial role in PA progression. On the other hand, miR‐106b was found to be significantly upregulated in IPAs and was shown to modulate the migratory and invasive capabilities of PA cells by regulating PTEN expression. This, in turn, alters the activity of the PI3K/AKT signaling pathway and ultimately impacts the expression of MMP‐9, further emphasizing the complexity and significance of miRNA regulation in PA biology [69].

#### 5.2.3 Long Noncoding RNAs (lncRNAs)

LncRNAs occupy a central position in numerous physiological and pathological processes, particularly in the initiation and progression of tumors. By intricately regulating the proliferation, migration, invasiveness, and metastatic abilities of tumor cells, lncRNAs have garnered attention as potential diagnostic and prognostic biomarkers for various tumor types, including PAs [70]. Wu et al. [71] observed that the expression of lncRNA BBOX 1‐AS1 was upregulated in PA tissues and cells. Importantly, when BBOX 1‐AS1 was downregulated, it suppressed PA cell invasion, apoptosis, and proliferation, while also inhibiting tumor growth in vivo [71]. Moreover, the knockdown of BBOX 1‐AS1 expression notably hindered tumor progression in animal models [71]. Another lncRNA, SNHG6, was found to be significantly elevated in IPA samples. This upregulation of SNHG6 enhanced the viability, migratory capacity, invasive potential, and epithelial‐to‐mesenchymal transition (EMT) process of PA cells [72]. LncRNA MYMLR was also shown to exhibit elevated expression in PA tissues compared to normal tissues. Knockdown of MYMLR reduced cell proliferation, migration, and invasion while inducing apoptosis in PA cells [73]. Furthermore, in vivo xenograft models demonstrated that MYMLR knockdown suppressed PA tumor growth [73]. A comprehensive transcriptome RNA sequencing (RNA‐seq) analysis identified LINC00473 as the most upregulated lncRNA in IPAs. This upregulation facilitated the cell cycle process, thereby promoting the proliferation of PA cells and contributing to the progression of IPAs [74]. These findings underscore the complex and multifaceted roles of lncRNAs in PA biology and their potential as therapeutic targets.

#### 5.2.4 Circular RNAs (circRNAs)

CircRNAs, in contrast to linear RNAs, exhibit a unique closed‐loop structure and encompass a broad spectrum of biological functions. Their exceptional stability, precision in timing, tissue specificity, and disease‐specific signatures have garnered considerable interest. Extensive research has illuminated circRNAs' pivotal roles in modulating tumor growth, invasion, metastasis, metabolic processes, immune responses, and other tumor‐related mechanisms [75]. A circRNA array analysis unveiled distinct differences in the circRNA landscape between NFPAs and normal pituitary tissues. Notably, circVPS13C was markedly overexpressed in NFPA samples and cell lines, and its silencing effectively suppressed pituitary tumor cell proliferation both in vitro and in vivo [76]. Clinically, elevated circVPS13C levels were observed in invasive NFPAs, while a decrease was noted in patient serum post‐transsphenoidal resection [76]. CircNFIX, another circRNA, was found to be overexpressed in IPAs, impacting tumor cell invasion, migration, and proliferation, suggesting its potential as a therapeutic target for PAs [77]. Furthermore, circDennd1b, abundant in exosomes derived from PA fibroblasts, was significantly upregulated in PAs. Its role in enhancing cell proliferation, migration, and invasion underscores its promise as a therapeutic strategy against aggressive PAs [78]. A comparative analysis revealed a distinctive circRNA expression pattern between invasive and noninvasive NFPAs, with 91 circRNAs upregulated and 61 downregulated in the invasive subtype [79]. Notably, hsa_circRNA_102597, a downregulated circRNA, displayed a significant correlation with tumor size and Knosp grade. This circRNA, either alone or in combination with the Ki‐67 index, exhibited promise in differentiating invasive from noninvasive NFPAs and predicting tumor progression/recurrence [79]. Additionally, circDDX17 downregulation in PA tissues was associated with invasion, tumor size, and progression‐free survival of PA patients, further emphasizing the significance of circRNAs in PA biology and prognosis [80]. Enhancing circDDX17 expression significantly impeded migration and invasion capabilities, indicating its function as a tumor suppressor and highlighting its potential as a valuable biomarker and promising therapeutic target for PA management [80].

### 5.3 Angiogenesis and Its Significance in IPAs

The vascular architecture of normal pituitary glands and PAs exhibits stark differences. While normal pituitary acini are nourished by a capillary network abundant in fenestrations, PAs typically display a suboptimal blood supply. Electron microscopic analysis uncovers that tumor vascular endothelial cells possess fewer fenestrations, accompanied by thickened and discontinuous basement membranes, highlighting the pivotal role of angiogenesis in PA development and its predictive value for tumor growth and metastasis [81]. On the positive side, the tumor vascular network supplies oxygen and nutrients and facilitates metabolite excretion, thereby fostering tumor expansion. However, the flawed vascular wall structure and irregular basement membrane thickness can also facilitate the hematogenous dissemination of tumor cells. The malignancy grade of many tumors is directly proportional to the abundance of tumor blood vessels, with highly aggressive tumors characterized by robust angiogenesis [82]. Intriguingly, PAs exhibit notably lower vascular densities compared to non‐tumorous adenohypophysis, contrasting with other organs. This suggests that the scarcity of substantial angiogenesis may contribute to the slow growth rate of pituitary tumors and their infrequent metastases, underscoring the unique biological behavior of these neoplasms.

Angiogenesis, a complex and multi‐staged process, involves a myriad of factors intricately orchestrating each phase of neovascularization. Among these, VEGF emerges as a pre‐eminent inducer of angiogenesis, playing a pivotal role not only in developmental processes but also in pathological conditions, particularly in pituitary tumors. VEGF is firmly established as a key player in PA tumorigenesis [83]. A proteomics and ingenuity pathway analysis has illuminated the significant association of the VEGF‐enriched pathway with the proliferation, migration, and invasion of invasive pituitary tumors [84]. Furthermore, research has demonstrated that pituitary tumor‐associated fibroblasts exhibiting cavernous sinus invasion secrete notably higher levels of VEGF compared to those from noninvasive tumors. This elevated VEGF secretion has been correlated with an increased capillary density, underscoring its crucial role in promoting angiogenesis and aggressive tumor behavior [85].

### 5.4 Cell Cycle/Proliferation Abnormalities and Their Association With IPAs

Proliferating cell nuclear antigen (PCNA), a vital cell cycle regulatory protein intimately linked to DNA replication and cell proliferation, stands as an objective and reliable marker for predicting the surgical prognosis of PAs [86, 87]. Notably, PCNA expression positively correlates with PA invasiveness, emphasizing its significance in tumor aggressiveness. Research has revealed that the mRNA level of PCNA was significantly upregulated in invasive NFPAs compared to their noninvasive counterparts. Intriguingly, this elevation inversely correlates with the mRNA levels of Smad3 [88] and TGF‐β RII [89], suggesting that heightened PCNA expression may facilitate the development and invasion of NFPAs. Consequently, PCNA emerges as a promising biomarker for diagnosing invasive NFPAs. Moreover, a comparative study has underscored significant statistical differences in both the growth rate and PCNA expression, advocating for the utilization of PCNA as an indicator to correlate with the progression of PA growth [90].

Ki‐67 serves as a highly sensitive and specific marker for assessing the proliferation index of human tumor cells, playing a pivotal role in predicting the prognosis of numerous neuroendocrine tumors, including PAs [91]. Numerous studies have underscored the robust association between Ki‐67 expression and PA invasiveness. Notably, a significantly higher Ki‐67 antigen level has been observed in pituitary neoplasms invading the sphenoidal sinus (SS), with a cut‐off point of 3.25% indicating an elevated risk for such invasion [92]. Furthermore, an observational single‐center study revealed that patients with a higher Ki‐67 index tend to exhibit elevated GH levels, enlarged tumor sizes, and a greater propensity for cavernous sinus invasion, particularly in somatotroph pituitary tumors [93]. A retrospective analysis has also emphasized the clinical relevance of Ki‐67 in predicting the aggressiveness of PA behavior. This study demonstrated a correlation between increased Ki‐67 levels, mitotic activity, and invasiveness, highlighting the significance of Ki‐67 in forecasting the progression of IPA behavior [94].

### 5.5 ECM Remodeling and Its Involvement in IPAs

Matrix metalloproteinases (MMPs), a class of zinc‐dependent proteolytic enzymes, play a pivotal role in regulating the ECM in both physiological and pathological conditions. These enzymes are crucial in the invasive and infiltrative growth of many malignant tumors, including their metastasis to distant tissues and organs [95]. MMPs accomplish this through three primary mechanisms: hydrolyzing the ECM surrounding tumor cells to break down the physical barrier; enhancing the adhesive forces between tumor cells and their surroundings to promote infiltrative growth; and interacting with ECM components to activate or stimulate other bioactive molecules that further facilitate tumor invasion [96]. Among MMPs, MMP‐14 has been notably upregulated in IPAs compared to noninvasive counterparts. Specifically, targeted silencing of MMP‐14 in TtT/GF PA cells significantly hindered cell migration, indicating its involvement in cavernous sinus invasion in PAs [97]. Therefore, MMP‐14 emerges as a potential diagnostic and therapeutic target for IPAs. Furthermore, IPAs exhibited increased MMP‐8 expression at both protein and mRNA levels, accompanied by decreased TIMP‐1 expression [98]. Correspondingly, patients with invasive adenomas had elevated serum MMP‐8 levels and lower TIMP‐1 levels [98], suggesting that these molecules serve as valuable markers for assessing IPA invasion. MMP‐9, another MMP family member, presents as a promising target for suppressing tumor immune evasion in PAs. Higher levels of MMP‐9 mRNA and protein have been observed in PAs compared to healthy tissues, where it facilitates the cleavage of MICA into soluble MICA, ultimately promoting tumor immune escape [99]. Furthermore, the MMP‐9 (−1562) C/C genotype plays a significant role not only in nonrecurrence, inactive, and invasive but also in the development of noninvasive PAs [100].

E‐cadherin, encoded by the *CDH1* gene, is a fundamental component of adherens junctions, essential for maintaining cell adhesion and epithelial cell identity. Its homophilic binding between cells is critical in mediating contact inhibition of proliferation, ensuring that cell growth is restrained once cells reach confluence. Loss of E‐cadherin expression disrupts this inhibition, enhancing cellular motility and frequently correlating with more aggressive cancer stages [101]. In pituitary tumors, a notable reduction in E‐cadherin expression has been observed in GH‐PAs compared to PRL‐PAs, as well as in IPAs and clinically recurrent PAs [102]. This downregulation is significantly associated with tumor subtype, invasiveness, and postoperative recurrence. Specifically, E‐cadherin expression was diminished in invasive NFPAs compared to their noninvasive counterparts, and its level negatively correlates with the Knosp classification, further emphasizing its link to invasiveness [103]. A similar trend has been observed in ACTH‐PAs, where decreased nuclear E‐cadherin expression and *CDH1* mRNA levels have been correlated with disease progression [104]. However, there remains some controversy in the literature, as one study found no direct relationship between E‐cadherin and invasiveness in NFPAs [105]. Additionally, a recent retrospective analysis of adult patients with ACTH‐PAs failed to uncover a significant correlation between E‐cadherin expression and tumor subtype, size, or prognosis [106].

## 6 Pharmacological Treatment Strategies for IPAs

The surgical objectives for PAs encompass tumor removal, alleviation of compression on adjacent anatomical structures, restoration of normal endocrine function, and prevention of recurrence. The extent of tumor resection achieved and the subsequent postoperative adjuvant therapies administered have a direct and profound impact on the therapeutic outcomes of IPAs. However, achieving complete surgical resection of IPAs is exceedingly challenging, and these tumors are associated with a high rate of postoperative recurrence. Consequently, drug therapy and additional adjunctive measures are frequently necessitated to manage the disease, posing a significant hurdle within the realm of contemporary neurosurgery.

### 6.1 Pharmacological Management of Invasive PRL‐PAs

Dopamine agonists (DAs) are the primary therapeutic drugs employed in the management of PAs, particularly PRL‐PAs. Traditionally, bromocriptine and cabergoline have been at the forefront of DA therapy for PRL‐PAs, with surgery often considered a secondary treatment option [107]. The mechanism underlying the efficacy of DAs involves their binding to specific dopamine type 2 receptors (D2) located on the cell membrane. This interaction inhibits PRL synthesis and secretion at the level of gene transcription, halts cell proliferation, and leads to cellular atrophy. In the context of PRL‐PAs treatment, DA therapy can result in the normalization of PRL levels, reduction in tumor volume, alleviation of galactorrhea symptoms, and even restoration of gonadal and fertility functions [108]. Furthermore, even when PRL‐PAs compress the optic chiasm, causing visual disturbances, DA treatment can effectively reduce tumor size, often meeting the patient's daily visual function requirements [109].

### 6.2 Pharmacological Treatment of Invasive GH‐PAs

Among functional PAs, the incidence of GH‐PAs ranks second only to PRL‐PAs. Notably, patients with high GH secretion exhibit a mortality rate several times higher than those with normal GH levels. While surgical resection of GH microadenomas can achieve a cure rate exceeding 80%, the effective control rate for invasive GH‐PAs through surgery alone is only approximately 30% [110]. Moreover, the long‐term surgical remission rate for GH‐PAs is notably lower than the initial “early” remission rates and heavily depends on the completeness of tumor resection [111]. Consequently, while surgery remains the preferred treatment option for GH‐PAs, it is prudent not to rely solely on surgical methods for invasive GH‐PAs, necessitating the combination of chemotherapy or radiotherapy. The objective of pharmacological therapy is to alleviate tumor compression symptoms, ameliorate symptoms arising from abnormal hormone secretion, and restore normal hormone levels in patients. Octreotide, a representative drug and a derivative of somatostatin, is primarily utilized in the treatment of GH‐PAs [112]. It effectively inhibits GH synthesis and secretion, suppresses tumor growth, and promotes tumor softening and size reduction before surgery, thereby facilitating surgical procedures and postoperative adjuvant therapy, as well as managing postsurgical hypersecretion of GH [112]. Studies have shown that octreotide normalized GH levels in two‐thirds of acromegaly patients and achieves significant tumor shrinkage [113].

### 6.3 Pharmacological Management of Invasive ACTH‐PAs

ACTH‐PAs constitute approximately 5%−10% of all functional PAs. Surgical resection of ACTH microadenomas can yield remission rates ranging from 60% to 90%, but tumor recurrence can compromise the effectiveness of surgery, with even lower success rates observed in invasive ACTH adenomas [114]. Currently, the pharmacological treatment for ACTH adenomas is primarily indicated for patients who do not respond to surgery, experience recurrence, or are unable to undergo surgical procedures. Drugs specifically targeting ACTH‐PAs have demonstrated efficacy, including cabergoline, a dopamine receptor type 2 agonist, and pasireotide, a somatostatin analog with multiple‐receptor activity [115]. Steroidogenesis inhibitors, like ketoconazole and metyrapone, provide rapid and sustained control for Cushing's disease caused by ACTH‐PAs [116]. Ketoconazole is particularly suitable for female patients and those without severe liver impairment, while levoketoconazole may offer a more potent alternative with reduced hepatotoxicity. Metyrapone is preferred for male patients and those without severe hypokalemia. Osilodrostat is ideal for long‐term treatment and patients with poor compliance. These steroidogenesis inhibitors can be administered alone or in combination with pituitary‐targeting drugs to enhance efficacy, reduce dosage requirements, and potentially minimize adverse event rates.

### 6.4 Pharmacological Treatment of Invasive GN‐PAs

The research landscape for drug‐based treatment of GN‐PAs remains largely exploratory. While several drug classes, such as DAs, somatostatin receptor ligands, and a GH receptor antagonist, show promise in altering the morphological features of GN‐PAs, their overall therapeutic effect is constrained [117]. In contrast to the pronounced efficacy seen in PRL‐PAs treated with DAs, a notable reduction in cell size is not a typical outcome in GH adenomas treated with DAs or somatostatin receptor ligands. Instead, the most prevalent changes involve varying degrees of perivascular and interstitial fibrosis [118]. Furthermore, at the ultrastructural level, there is an enlargement of secretory granules and the presence of enlarged, heterogeneous lysosomes that are actively engaged in the uptake of these granules. These alterations are believed to stem from the inhibition of hormone release.

### 6.5 Pharmacological Management of Invasive TSH‐PAs

Invasive TSH‐PAs are an uncommon occurrence in clinical settings, comprising roughly 1% of all PAs [119]. The gold standard for definitive treatment remains transsphenoidal surgery. Traditionally, radiation therapy was reserved as a secondary option for patients with residual or recurrent tumors following surgery. However, with the advent of somatostatin analogs, which possess the ability to normalize thyroid function and shrink tumors, medical therapy has gained significant traction for those who fail to achieve remission after pituitary surgery [120]. Additionally, DAs have shown moderate success in managing TSH‐PAs [121]. In cases where surgical intervention is unsuccessful, somatostatin analogs have proven effective in normalizing TSH secretion in over 90% of patients and reducing tumor size in more than 40% of patients, making them a valuable therapeutic option [122].

### 6.6 Chemotherapeutic Approaches for IPAs

Temozolomide (TMZ) exerts its antitumor effects primarily by inducing base mismatches during DNA replication, ultimately leading to tumor cell apoptosis. Additionally, TMZ's unique ability to readily cross the blood–brain barrier, inhibit tumor cell growth throughout the cell cycle, and demonstrate broad‐spectrum antitumor activity makes it a valuable therapeutic option [123]. Consequently, it is not only the first‐line chemotherapy drug for malignant gliomas but also holds significant promise for the treatment of IPAs [124]. TMZ can be administered to three specific patient populations: PRL‐PA patients who are unresponsive to bromocriptine or cabergoline or have experienced recurrence following surgery and radiation therapy; ACTH‐PA patients who have failed surgical and radiation treatments, particularly those with Crooke's cell adenomas and Nelson's syndrome; and NFPA patients who have experienced regrowth or recurrence after multiple surgeries and radiation treatments [124]. However, the long‐term efficacy of TMZ and the potential for stabilization after discontinuation remain unclear. While TMZ has paved a new avenue for treating IPAs, particularly those resistant to surgery, radiation, and conventional therapies, it is generally regarded as a salvage treatment for refractory pituitary tumors and carcinomas globally. There is a notable lack of large‐scale randomized controlled trials and long‐term follow‐up studies evaluating the effectiveness of TMZ. Furthermore, it is important to acknowledge that, despite its low incidence, chemical meningitis is a unique complication associated with TMZ treatment for IPAs [125]. For instance, during chemotherapy, certain medications can either stimulate or exert toxic effects on the meninges, eliciting an inflammatory response. This, in turn, can lead to the development of chemical meningitis, a condition characterized by a range of symptoms including persistent headaches, meningeal irritation signs, fever, nausea, vomiting, and potential neurological dysfunction [126]. The onset of these symptoms is a direct consequence of the medication's interaction with the meninges, highlighting the need for careful monitoring and management during chemotherapy treatment.

### 6.7 Molecularly Targeted Therapies for IPAs

Several novel targeted therapeutic drugs have emerged, including the mammalian target of rapamycin (mTOR) inhibitors, EGF receptor (EGFR) inhibitors, and drugs that target VEGF. Research has shown that the mTOR pathway is activated in PRL‐PAs, and in vitro studies have demonstrated the antiproliferative effects of everolimus, an mTOR inhibitor, suggesting its potential as a novel therapeutic approach for aggressive PRL‐PAs that do not respond to standard treatments [127]. Previous investigations into EGF and its receptor have fueled the study of tyrosine kinase inhibitors, particularly EGFR inhibitors like gefitinib. These targeted therapeutic drugs have shown promise in in vitro experiments on invasive ACTH‐PAs [128]. In a case study, Ortiz et al. [129] reported the long‐term control of an invasive ACTH‐PA after 26 months of treatment with the anti‐VEGF drug bevacizumab. Furthermore, a Phase I clinical trial explored the feasibility and safety of implanting Gliadel wafers, a slow‐release drug delivery system containing the chemotherapy agent carmustine, into the sella turcica of a select group of patients with aggressive PAs [130]. This approach aims to deliver high concentrations of carmustine directly to the tumor site, minimizing systemic exposure and potential side effects [130]. While molecular targeted therapy offers new avenues for addressing the challenge of treating IPAs with conventional drugs that are typically used for noninvasive PAs, the safety and efficacy of these studies have yet to be confirmed by extensive clinical trials.

### 6.8 Tumor Immunity in IPAs

The phenotype of unfavorable PAs is influenced not only by the intrinsic behavior of tumor cells but also by the immune cells infiltrating the TME [131]. Two key immune‐inhibitory checkpoints targeted in the TME are cytotoxic T‐lymphocyte antigen 4 (CTLA‐4) and programmed cell death protein 1 (PD‐1) (Table 4 [132, 133]). Notably, anti‐CTLA‐4 therapy has been the first immunotherapy approved to show survival benefits for patients with metastatic melanoma [134]. Enhancing our understanding of the immune characteristics within the TME and developing novel classifications based on distinct immune traits can offer insights into the mechanisms underlying varied immunotherapy responses and serve as a foundation for future targeted research endeavors [135]. Investigations into intratumoral T‐cell infiltration and the expression of programmed death‐ligand 1 (PD‐L1) in PAs have been conducted in several studies. PD‐L1 was frequently expressed in functioning PAs with the association of aggressive behaviors in PAs [131]. The majority of PAs consist of macrophages and T cells, with each subtype exhibiting a unique immune infiltration pattern [136]. CD68+ and CD8+ immune cells have been identified as factors correlated with the growth characteristics of somatotroph tumors and their responsiveness to first‐generation somatostatin analogs, suggesting that lymphocytes and macrophages form an immune network within somatotropinomas, and the features of the immune infiltrate have the potential to forecast treatment outcomes [137]. Also, a reduced number of CD8 + T cells are associated with both cavernous sinus invasion and treatment resistance in PAs [138]. Despite the above studies providing an initial glimpse into the distribution of tumor‐infiltrating immune cells (TIICs) in PAs, the comprehension of the immune profile in PAs remains limited, and the clinical significance of these immune patterns has yet to be fully explored. A recent study has revealed that the distributions of TIICs vary between PAs and normal pituitaries. Also, among different subtypes of PAs, T cells constitute the dominant component of the immune microenvironment across all PA subtypes; the abundance of TIICs is correlated with tumor size and patient age, while mutations in *USP8* in corticotroph adenomas have an impact on the intratumoral distribution of TIICs [139]. Based on the TIIC distribution, three immune clusters have also been distinguished among PAs [139]. These findings lay the groundwork for deeper immune research on IPAs and offer fresh perspectives on potential immunotherapy approaches for treating IPAs.

**Table 4:** The key immune checkpoint molecules and their main functions in IPAs.

Immune checkpoint molecules	Functions	Diseases
CTLA‐4	Overexpression of CTLA‐4 may inhibit the activity of antitumor T cells, thereby suppressing tumor immune response.	ACTH‐PAs PRL‐PAs
PD‐1	Overexpression of PD‐1 may lead to suppression of antitumor T‐cell function, thereby limiting the tumor immune response.	Cushing's disease ACTH‐PAs PRL‐PAs
PD‐L1	Binding of PD‐L1 to PD‐1 reduces the recognition and killing ability of T cells toward tumor cells, enabling immune evasion.	Cushing's disease ACTH‐PAs PRL‐PAs

## 7 Conclusion

Despite their benign nature, IPAs exhibit malignant biological traits, such as invading adjacent tissues, displaying invasive growth patterns, having low rates of complete surgical resection, experiencing high recurrence rates, and often requiring postoperative radiotherapy or drug therapy. Investigations into the invasiveness of PAs have uncovered a range of biological markers intimately linked to tumor initiation and progression. These markers not only shed light on the proliferation, invasion, and metastasis behaviors of PA cells but also present novel avenues for PA diagnosis and treatment. In diagnosing IPAs, two key aspects must be concurrently evaluated: the tumor's invasive growth pattern, discernible through preoperative imaging, intraoperative assessment, and postoperative pathological examination; and the tumor's invasive growth behavior, assessable via molecular biology, immunohistochemical studies, and recurrence patterns. Given the absence of a universally accepted international definition for IPAs, a comprehensive grading system that assigns varying weights to these factors and correlates them with clinical prognosis would be invaluable in guiding IPA treatment strategies. Surgical intervention remains the primary treatment modality for various IPA types, with drug therapy serving as a secondary option when surgery fails to adequately address hypersecretion. However, the drugs currently employed in IPA treatment are primarily conventional therapies for noninvasive PAs, with limited clinical breakthroughs. While TMZ and molecular targeted therapies have shown promising research outcomes in IPA treatment, their efficacy and safety profiles necessitate further exploration and clarification.

As biotechnology progresses, research methodologies continually evolve, and novel theories emerge, clinicians and researchers gain an increasingly comprehensive and nuanced understanding of PA invasiveness. This deepened comprehension not only enhances the accuracy of PA imaging and pathological diagnosis but also underscores the predictive power of molecular markers in assessing patient prognosis, holding significant implications for optimizing treatment outcomes for patients with IPAs. Furthermore, these insights serve as vital targets for the burgeoning realm of PA gene therapy, paving the way for the development of innovative treatment strategies in the future.

## Author Contributions

D.X. designed, wrote, and revised the manuscript. L.W. provided financial support. M.Z. retrieved literature and databases and revised and approved the manuscript.

## Conflicts of Interest

The authors declare no conflicts of interest.

## Data Availability

The authors have nothing to report.
